# Suppressed topological phase transitions due to nonsymmorphism in SnTe stacking

**DOI:** 10.1038/s41598-018-27827-x

**Published:** 2018-06-21

**Authors:** Augusto L. Araújo, Gerson J. Ferreira, Tome M. Schmidt

**Affiliations:** Instituto de Física, Universidade Federal de Uberlândia, Uberlândia, Minas Gerais 38400-902 Brazil

## Abstract

We combine first principles calculations with a group theory analysis to investigate topological phase transitions in the stacking of SnTe monolayers. We show that distinct finite stacking yields different symmetry-imposed degeneracy, which dictates the hybridization properties of opposite surface states. For SnTe aligned along the [001] direction, an (even) odd number of monolayers yields a (non)symmorphic space group. For the symmorphic case, the hybridization of surface states lead to band inversions and topological phase transitions as the sample height is reduced. In contrast, for a nonsymmorphic stacking, an extra degeneracy is guaranteed by symmetry, thus avoiding the hybridization and topological phase transitions, even in the limit of a few monolayers. Our group theory analysis provide a clear picture for this phenomenology and matches well the first principles calculations.

## Introduction

The recently discovered topological insulators (TIs) are classified accordingly to the symmetries of its crystal lattice and/or time-reversal symmetry, which yields its topologically protected edge or surface states^1,2^. Particularly, the class of topological crystalline insulators (TCIs) have Dirac-like bands protected by space group symmetries^3,4^. The first material predicted to be a TCI was SnTe^5^, which was promptly verified experimentally^6^, and followed by other IV-VI compounds^7–10^. The monolayers of IV-VI materials have been predicted to be two-dimensional (2D) TCIs^11,12^. Both the bulk and monolayers of SnTe are classified by their mirror Chern number |*n*_*M*_| = 2, yielding an even number of Dirac cones in the Brillouin zone. In between these two limits, the stacking of monolayers show an intriguing nonmonotonic evolution of the band gap for stackings oriented along the [001]^12–15^, [111]^16–20^ and [110]^21,22^ directions. Particularly, odd and even stackings of [001] SnTe belong to distinct space groups: the first is symmorphic, while the latter is nonsymmorphic; i.e. some symmetry operations require translations by a fraction of the unit cell. It is known that nonsymmorphic groups yield an extra degeneracy with respect to its symmorphic counterpart^23,24^. Moreover, from the nonsymmorphic space groups arise newly proposed exotic topological properties^25–32^.

In this paper the distinct topological phases of odd and even stackings of SnTe monolayers are explained by the contrast between their symmorphic and nonsymmorphic crystal symmetries. First, we analyze first principles [density functional theory (DFT)^33–36^, see Methods] band structures as a function of the number *N* of stacked layers. Interestingly, we find that the symmorphic stacking presents two topological phase transitions, while no phase transition is seen for the nonsymmorphic stackings. To understand this disparity, we develop low energy models for the surface states of both symmorphic (odd *N*) and nonsymmorphic (even *N*) stackings. We show that, due to finite-size effects, topological surface states located at opposite surfaces hybridize differently in the symmorphic and nonsymmorphic cases. Particularly, the nonsymmorphism of the even stackings enforces an extra degeneracy, thus avoiding a band inversion. Ultimately, this protects the system from a topological phase transition. Our results provide a clear picture of finite size effects, and its respective space group, on the hybridization of surface states. Although we focus here on the [001] stacking of SnTe monolayers, equivalent considerations shall hold for other directions, and as well for any material that may present distinct space groups for different stacking heights.

## Results and Discussions

### DFT analysis

**Figure 1:**
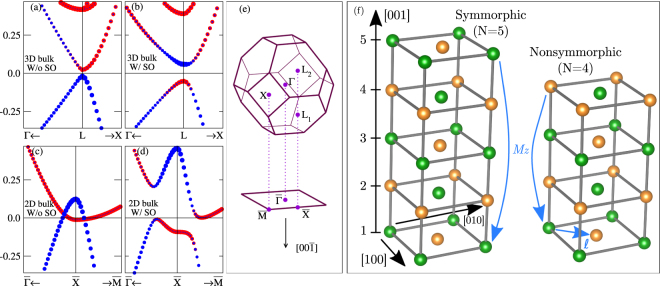
Band inversions for (**a**,**b**) bulk and (**c**,**d**) monolayer SnTe. Blue and red dots label are projections of ${{\rm{\Phi }}}_{+}=[{\phi }_{P}^{Te}+{\phi }_{S}^{Sn}]$ and ${{\rm{\Phi }}}_{-}=[{\phi }_{P}^{Sn}+{\phi }_{S}^{Te}]$, respectively. (**e**) 3D bulk Brillouin zone and its projection into the (001) surface. (**f**) Symmorphic (odd *N*) and nonsymmorphic (even *N*) stackings of SnTe monolayers. The blue arrows illustrate the mirror operation along z for the symmorphic (*M*_*z*_) and nonsymmorphic ($\{{M}_{z},\overrightarrow{\ell }\}$) cases.

Bulk SnTe has a rock-salt lattice and shows a small band gap at the L point. There, the conduction and valence bands are inverted due to the spin-orbit (SO) interaction, as shown in Fig. 1(a,b), yielding its 3D TCI regime characterized by the mirror Chern number |*n*_*M*_| = 2. Around the L point, the effective Hamiltonian^37^ obeys the $P\bar{{\rm{3}}}1{\rm{m}}\,({\rm{or}}\,{{\rm{D}}}_{3{\rm{d}}}^{1})$ symmorphic space group. In the opposite limit, a single monolayer of SnTe is a 2D TCI^4,5,24,37^. In this case, without SO the bands are already inverted, but it is metallic around the $\overline{{\rm{X}}}$ point of the 2D Brillouin zone, Fig. 1(c). The SO coupling opens the gap shown in Fig. 1(d), yielding the topological regime. The band structure around $\overline{{\rm{X}}}$ obeys the symmorphic space group Pmmm $({\rm{or}}\,{{\rm{D}}}_{2{\rm{h}}}^{1})$^24^. The spinful double group of both bulk and monolayer admits doubly degenerate irreducible representations (IRREPs) with odd and even inversion characters^38^, which can be characterized by the orbital projections ${{\rm{\Phi }}}_{-}=[{\phi }_{P}^{Sn}+{\phi }_{S}^{Te}]$ and ${{\rm{\Phi }}}_{+}=[{\phi }_{P}^{Te}+{\phi }_{S}^{Sn}]$, respectively^39^. These projections label the color code in Fig. 1(a–d) and illustrates the band inversions.

#### Finite Stackings

**Figure 2:**
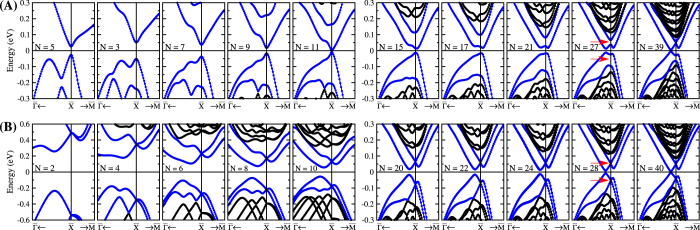
Band structure for SnTe stackings with *N* layers and ***k*** around the $\overline{{\rm{X}}}$ TRIM along the $\overline{{\rm{\Gamma }}}$ − $\overline{{\rm{X}}}$ and $\overline{{\rm{X}}}$ − $\overline{{\rm{M}}}$ directions [see Fig. 1(e)]. (**A**) The top row show the results for odd *N*, which corresponds to the symmorphic lattice symmetry, while (**B**) in the bottom row we show the even *N* nonsymmorphic case. The blue bands are colored to guide the eye throughout the hybridization of the surface states as *N* is reduced. The red arrows on the *N* = 27 and *N* = 28 panels illustrate the extra degeneracy imposed by the nonsymmorphic symmetry.

**Figure 3:**
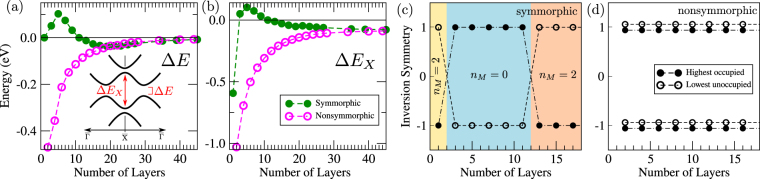
(**a**) Total energy band gap Δ*E* and (**b**) the local gap Δ*E*_*X*_ at $\overline{{\rm{X}}}$ [see inset of panel (**a**)] as a function of the number of layers *N*. (**c)** and (**d**) Inversion eigenvalues of the highest occupied (solid circles) and lowest unoccupied (empty circles) states from the DFT data for the symmorphic and nonsymmorphic stackings, respectively. In (**c**) the mirror Chern number *n*_*M*_ is indicated by the shade colors. For the symmorphic case two phase transitions are apparent, while for the nonsymmorphic stacking the fourfold degeneracy at $\overline{{\rm{X}}}$ avoids any phase transitions.

At the intermediate nanosize regime, composed by a finite number *N* of SnTe monolayers stacked along [001], Fig. 1(f), finite-size effects arise distinctively for odd or even *N*. In both cases, the stacking projects a pair of distinct L points into $\overline{{\rm{X}}}$, Fig. 1(e). For large *N*, the distinction between odd or even number of layers is small. Each surface shows a pair of Dirac bands, which are highlighted by the colors in Fig. 2(a,b). However, as *N* is reduced, topological states localized at opposite surfaces hybridize differently for the odd/even *N* (symmorphic/nonsymmorphic). The hybridization becomes relevant for stackings $\lesssim 4\lambda$, where $\lambda \sim \hslash {v}_{F}/{E}_{g}$ is the penetration length of the surface states. From Fig. 1(b) we estimate the bulk gap *E*_*g*_ ≈ 0.11 eV and Fermi velocity ℏ*v*_*F*_ ≈ 2 eVÅ, which gives *λ* ≈ 18 Å or *N* ≈ 6 layers. Therefore, the hybridization becomes relevant for $N\lesssim 24$, which matches the gap evolution in Fig. 3(a,b).

Next we first present these gaps and the inversion eigenvalues obtained from the DFT simulations for the symmorphic and nonsymmorphic cases for various *N*. Later on we introduce a group theory model for the surface states, which allow us to understand how the states from opposite surfaces hybridize as *N* is reduced. We find that the nonsymmorphic symmetry of even *N* stackings imposes an extra degeneracy at $\overline{{\rm{X}}}$, which protects the band crossings for all even *N*; e.g. see arrows in Fig. 2(b) for *N* = 28. In contrast, for odd *N* these bands hybridize as *N* is reduced; e.g. see arrows in Fig. 2(a) for *N* = 27. Within the model we will see that these distinct behaviors explain the phase transitions seen for the symmorphic case, and its absence for the nonsymmorphic stacking.

#### Symmorphic stacking

The *ab-initio* band structure for odd stacking ranging from *N* = 5 to 39 layers is shown in Fig. 2(a). For large *N*, the Dirac crossings that characterizes the TCI regime are noticeable (highlighted in blue). As *N* is reduced, the hybridization between the top and bottom surface states take place and the bands become massive. To analyze this process, we plot the total gap Δ*E* and the local gap Δ*E*_*X*_ at $\overline{{\rm{X}}}$ in Fig. 3(a,b). For the symmorphic case (solid green circles) two phase transitions are striking. That is, Δ*E*_*X*_ changes sign from *N* = 1 to 3, and from *N* = 11 to 13, which is characterized by the inversion eigenvalue in Fig. 3(c). For *N* = 1 the monolayer is a 2D TCI, in agreement with previous results^4,5,24,37^.

**Figure 4:**
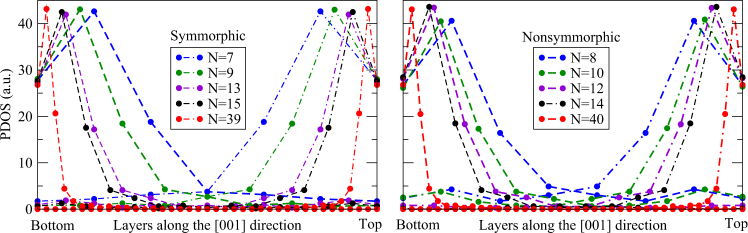
Projected density of sates (PDOS), at each layer along the [001] direction, for the surface states for different *N* stackings. States localized at the bottom (top) layer are drawn with dashed (dot-dashed) lines. Along the lines, each circle corresponds to a layer, which are normalized between the bottom and top layers. In all cases, the PDOS show that the densities are localized within $\lesssim 6$ layers from the surfaces.

To understand the transition from *N* = 11 to 13, the density of states for the surface bands around the Dirac crossing off the $\overline{{\rm{X}}}$ point are projected along the [001] layers, as shown in Fig. 4. Interestingly, in all cases (symmorphic and nonsymmorphic for all *N*) it shows that the densities are localized at the top and bottom surfaces, within the length *λ* (≈6 layers) as discussed previously. Each surface state belongs to an even or odd inversion IRREP of the Pmmm $({\rm{or}}\,{{\rm{D}}}_{2{\rm{h}}}^{1})$ space group^38^. These surface states do not change their character when passing from *N* <11 to> 13. In fact, the sole effect of reducing *N* is to increase the overlap and hybridization between top and bottom states. Therefore, for any $N\lesssim 6$ the staking maintains its 3D TCI character defined by its bulk invariants. Consequently, the band inversions observed for odd stacking shown in Fig. 3(c) must be of a lower order, i.e. a 2D topological phase transition. An illustrative model^40^ for the edge states is shown in the Supplementary Information. This 2D topological phase transition, can be better characterized by the mirror Chern number computed using the surface state bands. Using the effective model described below, we calculate this mirror Chern number *n*_*M*_ as a function of the gap sign (Δ*E*_*X*_). We find |*n*_*M*_| = 2 for Δ*E*_*X*_ < 0 and *n*_*M*_ = 0 for Δ*E*_*X*_ > 0. This is shown by the color code in Fig. 3(c). For the monolayer case (*N* = 1) it is known that |*n*_*M*_| = 2^12,24^.

### Nonsymmorphic stacking

For nonsymmorphic stackings we have a different picture. Both Δ*E* and Δ*E*_*X*_ show smooth monotonic and asymptotic behaviors in Fig. 3(a,b) (open pink circles). There is no phase transition. The band structures in Fig. 2(b) show clear hybridized Dirac bands for *N* > 6. Notice that the crossings at $\overline{{\rm{X}}}$ remain closed for all even *N*, in contrast with the hybridization seen in Fig. 2(a) for odd *N* (c.f. arrows for *N* = 27 and 28). For large *N* the nonsymmorphic stacking is indistinguishable from the symmorphic one, since finite size effects become negligible as the surface states are spatially split [c.f. *N* = 39 in Fig. 2(a), and *N* = 40 in Fig. 2(b)], yielding gapless Dirac crossings. As we will see later on, the lack of phase transitions in this case is due to the degeneracies imposed by the nonsymmorphic symmetry. Its space group is the Pmmn $({\rm{or}}\,{{\rm{D}}}_{2{\rm{h}}}^{13})$, and due to the Bloch phase at $\overline{{\rm{X}}}$, the only allowed double group IRREPs are degenerate due to time-reversal symmetry^24,38^. This yields a fourfold degeneracy with pairs of states with even and odd inversion eigenvalues. This is illustrated in Fig. 3(d). Moreover, from the PDOS shown in Fig. 4 we observe that the 3D topological character is always present for nonsymmorphic stacking, independent on the number of layers. Since there is no phase transition, its topological nature is governed by the precursor bulk 3D TCI with mirror Chern number |*n*_*M*_| = 2.

### Effective model

Next, we develop low energy models for symmorphic and nonsymmorphic stackings, which will help us to understand the phase transitions presented above. Indeed we find that only two transitions should be expected for the symmorphic case, in agreement with *ab-initio* results.

Let us start by defining a basis as the surface states from a semi-infinite stacking limit. Then, we allow it to hybridize with the solutions from the opposite surface due to finite symmorphic or nonsymmorphic stackings. In all cases we consider the wave-functions around $\overline{{\rm{X}}}$ to be composed by *s*, *p*_*x*_ and *p*_*z*_ atomic orbitals of Sn and Te, as obtained from the DFT first principles calculations.

#### Semi-infinite stacking

Around $\overline{{\rm{X}}}$, the semi-infinite stacking obeys the symmorphic Pmm2 (or C_2v_) space group. Starting with the spinless case, we find by inspection that the surface states belong to the *A*_1_ and *B*_1_ IRREPs of C_2v_. Therefore we label the spinful eigenkets for our basis as {*A*_1_; ↑, *A*_1_; ↓, *B*_1_; ↑, *B*_1_; ↓}, where {↑, ↓} refers to the spin along z. The C_2v_ group is composed by a rotation *C*_2_(*z*) = *iτ*_3_⊗*σ*_*z*_ around the *z* axis, and mirrors *M*_*x*_ = −*iτ*_3_ ⊗ *σ*_*x*_ and *M*_*y*_ = −*iτ*_0_ ⊗ *σ*_*y*_ that reflect *x* → − *x*, and *y* → − *y*, respectively. Here the *τ*_*i*_ (*i* = 0, 1, 2, 3) are su(2) matrices acting on the A_1/B_1 orbitals and *σ*_*j*_ (*j* = 0, *x*, *y*, *z*) are the su(2) spin matrices. The time-reversal symmetry operator is ${\mathscr{T}}=i{\tau }_{0}\otimes {\sigma }_{y}{\mathscr{K}}$, where ${\mathscr{K}}$ is the complex conjugation operator. Up to linear order in *k*_*x*_ and *k*_*y*_ the effective Hamiltonian that commutes with these symmetries is

1 $${H}_{\infty }=(\begin{array}{cccc}{e}_{0}+{{\rm{\Delta }}}_{0} & i{\boldsymbol{\alpha }}\cdot {{\bf{k}}}_{-} & i{\boldsymbol{\gamma }}\cdot {{\bf{k}}}_{-} & {{\rm{\Delta }}}_{1}\\ -i{\boldsymbol{\alpha }}\cdot {{\bf{k}}}_{+} & {e}_{0}+{{\rm{\Delta }}}_{0} & -{{\rm{\Delta }}}_{1} & i{\boldsymbol{\gamma }}\cdot {{\bf{k}}}_{+}\\ -i{\boldsymbol{\gamma }}\cdot {{\bf{k}}}_{+} & -{{\rm{\Delta }}}_{1} & {e}_{0}-{{\rm{\Delta }}}_{0} & i{\boldsymbol{\beta }}\cdot {{\bf{k}}}_{-}\\ {{\rm{\Delta }}}_{1} & -i{\boldsymbol{\gamma }}\cdot {{\bf{k}}}_{-} & -i{\boldsymbol{\beta }}\cdot {{\bf{k}}}_{+} & {e}_{0}-{{\rm{\Delta }}}_{0}\end{array}),$$

where ***k***_±_ = (*k*_*x*_, ±*ik*_*y*_). The coefficients ***α*** = (*α*_*x*_, *α*_*y*_) and ***β*** = (*β*_*x*_, *β*_*y*_) are in-plane (*σ*_*x*_ and *σ*_*y*_) SOCs that define the anisotropic Fermi velocity near ***k*** = 0 for the Dirac cones at energies $E={e}_{0}\pm \sqrt{{{\rm{\Delta }}}_{0}^{2}+{{\rm{\Delta }}}_{1}^{2}}$, while ***γ*** = (*γ*_*x*_, *γ*_*y*_) couples orbitals *A*_1_ to *B*_1_ with the same spin at finite ***k***.

**Figure 5:**
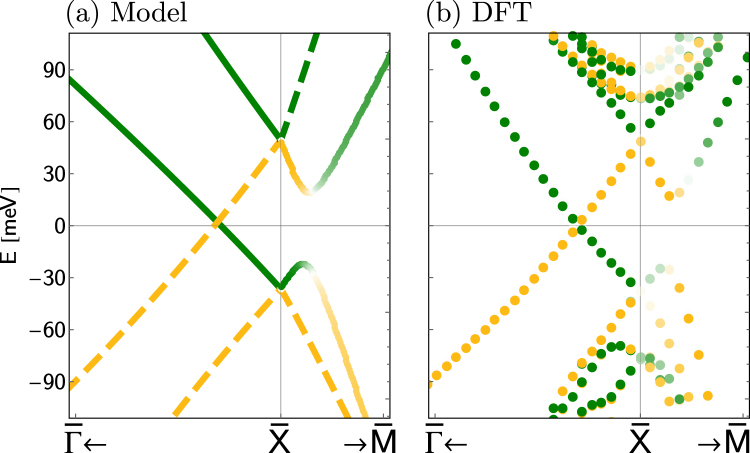
(**a**) Effective model for a semi-infinite stacking fitted to (**b**) the DFT data for a *N* = 60 monolayer stacking. Along the $\overline{{\rm{\Gamma }}}-\overline{{\rm{X}}}$ direction, both the mirror *M*_*y*_ = ±*i* (solid/dashed) and spin *σ*_*y*_ = {↑_*y*_, ↓_*y*_} (green/yellow) along *y* are equivalent. On the other hand, along $\overline{{\rm{X}}}-\overline{{\rm{M}}}$ only the mirror *M*_*x*_ = ±*i* (solid/dashed) is a good quantum number, while the spin *σ*_*x*_ = {↑_*x*_, ↓_*x*_} projection is labeled by the color gradient (from green to yellow). The agreement with the DFT data from panel (**b**) is clear.

We fit this Hamiltonian to the DFT data for a large stacking (*N* = 59 or 60), which gives the parameters: *e*_0_ = 0.0065 eV, Δ_0_ = 0.043 eV, Δ_1_ ≈ 0, *α*_*x*_ = 1.6 eV, *α*_*y*_ = 3.3 eV, *β*_*x*_ = 1.3 eV, *β*_*y*_ = 2.0 eV, *γ*_*x*_ = 0.4, *γ*_*y*_ = 1.5 eV. The resulting band structure is shown in Fig. 5. For small ***k*** near $\overline{{\rm{X}}}$ the agreement is patent, while for large ***k*** parabolic terms would be necessary to improve it. Particularly, along the $\overline{{\rm{X}}}-\overline{{\rm{M}}}$ direction one branch enters the conduction band quickly and can be disregarded, as we do not account for its coupling to the bulk in the effective model. More importantly, the spin projections (color coded) match well the DFT results. Along $\overline{{\rm{\Gamma }}}-\overline{{\rm{X}}}$, both the mirror *M*_*y*_ and the spin *σ*_*y*_ are preserved, allowing us to label the states with its eigenvalues *M*_*y*_ = ±*i*, which match the *σ*_*y*_ projections since [*M*_*y*_,*σ*_*y*_] = 0. Along $\overline{{\rm{X}}}-\overline{{\rm{M}}}$ the mirror *M*_*x*_ is preserved, while [*H*_∞_, *σ*_*x*_] ≠ 0, thus we label the states with the eigenvalues of *M*_*x*_ = ±*i*, while the spin *σ*_*x*_ projections are mixed (color code) at the anti-crossing induced by the *γ*_*y*_ term.

#### Finite stackings and phase transitions

The space groups of the symmorphic (${{\rm{D}}}_{2{\rm{h}}}^{1}$) and nonsymmorphic (${{\rm{D}}}_{2{\rm{h}}}^{13}$) stackings share the C_2v_ as a common subgroup. Therefore, to model the finite stackings, we consider that the top (*t*) and bottom (*b*) surface states are approximately given by the semi-infinite solutions above. That is, our basis is now labeled by $\{{A}_{1}^{t};\uparrow ,{A}_{1}^{t};\downarrow ,{B}_{1}^{t};\uparrow ,{B}_{1}^{t};\downarrow ,{A}_{1}^{b};\uparrow ,{A}_{1}^{b};\downarrow ,{B}_{1}^{b};\uparrow ,{B}_{1}^{b};\downarrow \}$. Introducing *s*_*j*_ (*j* = 0, 1, 2, 3) as su(2) matrices acting on the top/bottom subspace, the C_2v_ operations are now *C*_2_(*z*) = *is*_0 _⊗ *τ*_3 _⊗ *σ*_*z*_, *M*_*x*_ = −*is*_0_ ⊗ *τ*_3_ ⊗ *σ*_*x*_ and *M*_*y*_ = −*is*_0 _⊗ *τ*_0 _⊗ *σ*_*y*_. For the odd symmorphic stacking, the ${{\rm{D}}}_{2{\rm{h}}}^{1}$ also contains the operations that connect the top and bottom surfaces: inversion *I*, mirror *M*_*z*_, and rotations *C*_2_(*x*) and *C*_2_(*y*). On the other hand, for an even number of monolayers, the ${{\rm{D}}}_{2{\rm{h}}}^{13}$ space group is given by the C_2v_ operations, plus the nonsymmorphic counterparts of *I*, *M*_*z*_, *C*_2_(*x*) and *C*_2_(*y*). Using Seitz notation, these are $\{I,\overrightarrow{\ell }\}$, $\{{M}_{z},\overrightarrow{\ell }\}$, $\{{C}_{2}(x),\overrightarrow{\ell }\}$, $\{{C}_{2}(y),\overrightarrow{\ell }\}$, where $\overrightarrow{\ell }$ is a nonprimitive translation by half a unit cell [see Fig. 1(f)]. Since *M*_*z*_ = *I* ⋅ *C*_2_(*z*), *C*_2_(*x*) = *I* ⋅ *M*_*x*_, and *C*_2_(*y*) = *I* ⋅ *M*_*y*_ (and equivalent expressions for the nonsymmorphic operations), it is sufficient to define how the inversion operator acts on our basis in both cases. For the odd stacking, the inversion transforms ${A}_{1}^{t}\rightleftharpoons {A}_{1}^{b}$ and ${B}_{1}^{t}\rightleftharpoons {B}_{1}^{b}$, thus *I* = *s*_1 _⊗ *τ*_0_ ⊗ *σ*_0_. In contrast, due to the nonprimitive translation, the $\{I,\overrightarrow{\ell }\}$ transforms ${A}_{1}^{t}\rightleftharpoons {B}_{1}^{b}$ and ${A}_{1}^{b}\rightleftharpoons {B}_{1}^{t}$, which yields $\{I,\overrightarrow{\ell }\}={s}_{1}\otimes {\tau }_{1}\otimes {\sigma }_{0}$.

**Figure 6:**
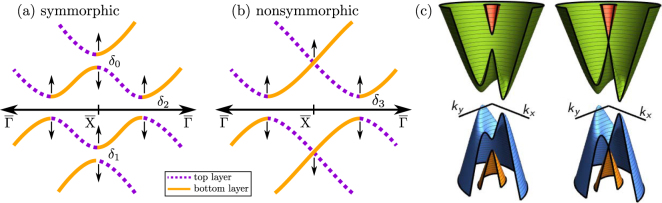
Illustration of the surface states dispersions along $\overline{{\rm{\Gamma }}}-\overline{{\rm{X}}}$ (i.e. *k*_*y*_ = 0) for the (**a**) symmorphic and (**b**) nonsymmorphic stackings within the *M*_*y*_ =  +*i* subspace. Due to the finite stacking, semi-infinite solutions from the top (purple, dashed) and bottom (orange, solid) layers hybridize, yielding the anticrossings *δ*_(0|1|2|3)_. The arrows indicate how each *δ*_*j*_ opens as one reduces the number of monolayers. These are degenerate with the *M*_*y*_ = −*i* subspace. (**c**) Energy dispersion as a function of ***k*** around $\overline{{\rm{X}}}$ extracted from the model for the symmorphic and nonsymmorphic stackings, respectively.

From the symmetries above, we construct the effective Hamiltonians up to linear order in ***k***. Its full form is shown in the Supplementary Information. However, to understand the topological phase transitions seen in the DFT results, it is sufficient to analyze the $\overline{{\rm{\Gamma }}}-\overline{{\rm{X}}}$ direction (i.e. *k*_*y*_ = 0). Along this direction *M*_*y*_ is a good quantum number, therefore we can block diagonalize the Hamiltonian into *M*_*y*_ = ±*i* subspaces. For *M*_*y*_ = + *i*, the dominant terms are

2 $${H}_{+i}^{(S)}\approx (\begin{array}{cccc}{{\rm{\Delta }}}_{0}-{\alpha }_{x}{k}_{x} & {\delta }_{0} & 0 & i{\delta }_{2}\\ {\delta }_{0} & {{\rm{\Delta }}}_{0}+{\alpha }_{x}{k}_{x} & i{\delta }_{2} & 0\\ 0 & -i{\delta }_{2} & -{{\rm{\Delta }}}_{0}-{\beta }_{x}{k}_{x} & {\delta }_{1}\\ -i{\delta }_{2} & 0 & {\delta }_{1} & -{{\rm{\Delta }}}_{0}+{\beta }_{x}{k}_{x}\end{array}),$$

3 $${H}_{+i}^{(NS)}\approx (\begin{array}{cccc}{{\rm{\Delta }}}_{0}-{\alpha }_{x}{k}_{x} & 0 & 0 & {\delta }_{3}\\ 0 & {{\rm{\Delta }}}_{0}+{\alpha }_{x}{k}_{x} & {\delta }_{3} & 0\\ 0 & {\delta }_{3} & -{{\rm{\Delta }}}_{0}-{\beta }_{x}{k}_{x} & 0\\ {\delta }_{3} & 0 & 0 & -{{\rm{\Delta }}}_{0}+{\beta }_{x}{k}_{x}\end{array}),$$

for the symmorphic [${H}_{+i}^{(S)}$] and nonsymmorphic [${H}_{+i}^{(NS)}$] cases. The effective Hamiltonians for *M*_*y*_ = −*i* are obtained simply replacing *α*_*x*_ → − *α*_*x*_ and *β*_*x*_ → − *β*_*x*_. In the semi-infinite limit, *α*_*x*_, *β*_*x*_ and Δ_0_ are the same as in *H*_∞_, but deviations should be expected for a small *N*. The new terms *δ*_*j*_ (*j* = 0, 1, 2, 3) represent the hybridization between top and bottom surface states, which couple the linear dispersions due to the finite size of the system. As the number of layers *N* is reduced, these hybridizations induce anticrossings *δ*_*j*_, which increase as indicated by the arrows in Fig. 6(a,b). Other terms allowed by symmetry only contribute to the fine tuning of the spectrum (see Supplementary Information). Three-dimensional visualizations of spectrums *E*(*k*_*x*_, *k*_*y*_) are shown in Fig. 6(c) using the parameters from the semi-infinite model above, and *δ*_0_ = *δ*_1_ = *δ*_2_ = 5 meV for the symmorphic stacking, and *δ*_3_ = 5 meV for the nonsymmorphic case. A direct comparison show that the only qualitative difference between the dispersions are the hybridization at $\overline{{\rm{X}}}$. For *δ*_1_ ≈ *δ*_0_, the gaps at $\overline{{\rm{X}}}$ [see inset of Fig. 3(a)] are

4 $${\rm{\Delta }}{E}_{X}^{(S)}\approx -\,2\sqrt{{{\rm{\Delta }}}_{0}^{2}+{\delta }_{2}^{2}}+2{\delta }_{0},$$

5 $${\rm{\Delta }}{E}_{X}^{(NS)}=-\,2\sqrt{{{\rm{\Delta }}}_{0}^{2}+{\delta }_{3}^{2}},$$

for the symmorphic and nonsymmorphic cases, respectively.

For the nonsymmorphic stacking, Eq. (5) tell us that the gap at $\overline{{\rm{X}}}$ is always ${\rm{\Delta }}{E}_{X}^{(NS)} < 0$ for any intensity of the finite-size hybridization *δ*_3_. Consequently, there cannot be an inversion of its surface state bands. Hence, there is no phase transition for the even stacking. This is a direct consequence of the fourfold degeneracy imposed by the nonsymmorphic symmetry, which does not allow the band crossing at $\overline{{\rm{X}}}$ to hybridize in Fig. 6(b). On the other hand, for the symmorphic stacking, the gap at $\overline{{\rm{X}}}$, given by Eq. (4), changes sign with increasing *δ*_0_ ≈ *δ*_1_, with the crossing point at ${\delta }_{0}\approx {\delta }_{1}=\sqrt{{{\rm{\Delta }}}_{0}^{2}+{\delta }_{2}^{2}}$, which corresponds to the *N* = 11 transition in Fig. 3(b). In both cases, we diagonalize the inversion operator within a degenerate subspace. The resulting eigenvalues match the DFT data in Fig. 3(c,d). Moreover, this hybridization picture agrees well with the DFT band structures from Fig. 2, and the gap evolutions shown in Fig. 3(a,b) for *N* ≥ 3.

The transition from *N* = 3 to 1 is more subtle. Indeed for a single monolayer (*N* = 1) the definition of top and bottom surfaces become meaningless. In order to understand this transition, first let us recall that the penetration length of the surface states throughout the bulk is $\lambda \sim 18$ Å or $N\sim 6$ layers. Therefore, for $N\lesssim 6$ we can safely assume that states from opposite surfaces hybridize, but does not reach all the way to the opposite surface, as seen in Fig. 4. This validates the model described above and the hybridization picture from Fig. 6. However, for $N\sim 6$ the surface states lengths are larger than the stacking itself, which now goes beyond our hybridization model above. Instead, we find that as *N* approaches the monolayer limit, a structural phase transition occurs. This can be characterized by the lattice parameters *a*_*N*_. For the monolayer we obtain *a*_1_ = 6.19 Å, while for *N* = 2 it jumps to *a*_2_ = 6.31 Å, and for *N* = 3, *a*_3_ = 6.33 Å. For larger *N* it smoothly evolves to the bulk value *a*_∞_ = 6.40 Å. This sharp transition from *N* = 3 to *N* = 1 enhances the atomic orbital hybridizations and atomic spin-orbit couplings, which dictates this phase transition.

## Conclusions

In conclusion, we have shown that the band structure of the symmorphic and nonsymmorphic stackings evolve differently as a function of *N*. This illustrates how the specific space group of a finite size sample is essential to understand its properties within the nanometer scale. Particularly, we show that the nonsymmorphic symmetry imposes an extra protected degeneracy that avoids an hybridization at the $\overline{{\rm{X}}}$ point, thus yielding a smooth evolution of the band structure with the number of layers. Consequently, there is no phase transition in this case, and its topological nature is governed by the precursor bulk 3D TCI regime, even for samples consisted of only a few layers. In contrast, the hybridization of the symmorphic stacking yields two 2D phase transitions. One is due to a band inversion induced by the hybridization, and the other occurs as the system approaches the monolayer limit. Our space-group-dependent hybridization picture provides a clear understanding of these two cases and matches well the DFT data. Moreover, this analysis can be easily extended to other finite-sized materials that may present a variety of distinct space groups.

## Methods

### Density functional theory

The first principles calculations are performed using the density functional theory (DFT) within the generalized gradient approximation (GGA) for the exchange and correlation functional^35^. Fully relativistic *j*-dependent pseudopotential within the projector augmented wave method^36^ has been used in the non-colinear spin-DFT formalism self-consistently. We use the Vienna Ab initio Simulation Package (VASP)^33,34^, with plane wave basis set with a cut-off energy of 400 eV. The Brillouin zone is sampled by using a number of k-points such that the total energy converges within the meV scale. All stackings have been fully relaxed. The optimized force criteria for convergence was less than 0.01 eV/Å. A lattice parameter of 6.19 Å was obtained for the monolayer, and 6.31 Å for the bilayer. For larger stackings, the lattice parameter varies smoothly as a function of the number of layers, and converges to the bulk value (6.40 Å) for $\lesssim 20$ layers.

### Group theory analysis

The effective models were built using the method of invariants^23,24,41^. In each case we consider the little group ${\mathscr{G}}$ that keeps the TRIM of interest invariant. To define the relevant irreducible representations (IRREPs) for our basis, we consider the orbitals that contribute to band structure around the Fermi level, as obtained in the DFT simulation. Matrix representations for each IRREP can be found by inspection. The spin degree of freedom is then included via a direct product with its su(2) operators to form the double group^38^. The character tables for each double group are shown in the Supplementary Information.

To obtain the effective Hamiltonians we consider the reducible representation composed by the direct sum of the IRREPs that define the chosen basis. For a generic Taylor expansion of *H*(***k***) as

6 $$H(k)=\sum _{n,m}{H}_{n,m}{k}_{x}^{n}{k}_{y}^{m},$$

we impose that *H*(***k***) commutes with all symmetry elements *S*_*i*_ of the group ${S}_{{i}}=\{{S}_{0},{S}_{1},{S}_{2},\ldots \}$. This constrains the matrices *H*_*n*,*m*_, thus yielding which of its matrix elements can be finite.

For the symmorphic lattices, it is sufficient to analyze its point group ${\mathscr{P}}={\mathscr{G}}/ {\mathcal B}$, which is the factor group of the full space group ${\mathscr{G}}$ with respect to the invariant subgroup of Bloch translations ${\mathcal B}$. In contrast, for the nonsymmorphic groups the factor group ${\mathcal F} ={\mathscr{G}}/ {\mathcal B}$ is not a point group. Instead, it contains elements of the form $\{{S}_{i}|{\ell }_{i}\}$ (Seitz notation), where *S*_*i*_ is a point group operation and ${\ell }_{i}$ is a fractional translation of the unit cell. Its action on a coordinate ***r***, its multiplication rule, and its matrix representations are^38^

7 $$\{{S}_{i}|{{\boldsymbol{\ell }}}_{i}\}{\boldsymbol{r}}={S}_{i}{\boldsymbol{r}}+{\ell }_{i},$$

8 $$\{{S}_{i}|{{\boldsymbol{\ell }}}_{i}\}\{{S}_{j}|{{\boldsymbol{\ell }}}_{j}\}=\{{S}_{i}\cdot {S}_{j}|{S}_{i}{{\boldsymbol{\ell }}}_{j}+{{\boldsymbol{\ell }}}_{j}\},$$

9 $${D}^{{\rm{\Gamma }}}(\{{S}_{j}|{\ell }_{j}\})={D}^{{\rm{\Gamma }}}({S}_{j}){e}^{i{\boldsymbol{k}}\cdot {\ell }_{j}},$$

where Γ refers to a given IRREP. Equation (9) shows that the matrix representation is the product of the corresponding point group matrix representation and a Bloch factor for the fractional translation ${\ell }_{j}$ for the point ***k*** of the Brillouin zone.

## Electronic supplementary material

Supplementary Information
