# Supplementary material for: Suppressed topological phase transitions due to nonsymmorphism in SnTe stacking
Source: Sci Rep. 2018 Jun 21;8:9452. doi: 10.1038/s41598-018-27827-x (PMC6013480; doi:10.1038/s41598-018-27827-x)
Supplement: Supplementary file 1 — Supplementary Information [file 41598_2018_27827_MOESM1_ESM.pdf]

Supplementary information for

# Suppressed topological phase transitions due to nonsymmorphism in SnTe stacking

**Augusto L. Araújo, Gerson J. Ferreira, and Tome M. Schmidt**

Instituto de Física, Universidade Federal de Uberlândia, Uberlândia, Minas Gerais 38400-902, Brasil

## Orbital arrangement

The DFT results tell us that the conduction and valence bands of SnTe are composed by $s$, $p_x$, and $p_z$ orbitals of both atoms, Sn and Te. Moreover, since we are interested in the band structure near the $\boldsymbol{k} = \bar{X} = \frac{\pi}{a}\hat{x}$ point of the Brillouin Zone, the Bloch theorem implies $\psi(x+a,y,z) = e^{ik_x a}\psi(x,y,z) = -\psi(x,y,z)$, where $a$ is the unit cell length along $\hat{x}$. Considering the irreducible representations (IRREPs) of the $C_{2v}$ group (semi-infinite stacking), these orbitals can be arranged as shown in Fig. 1. Notice that they obey the Bloch phase above, and the $C_{2v}$ IRREPs (Tab. 2) are labeled on top of each case.

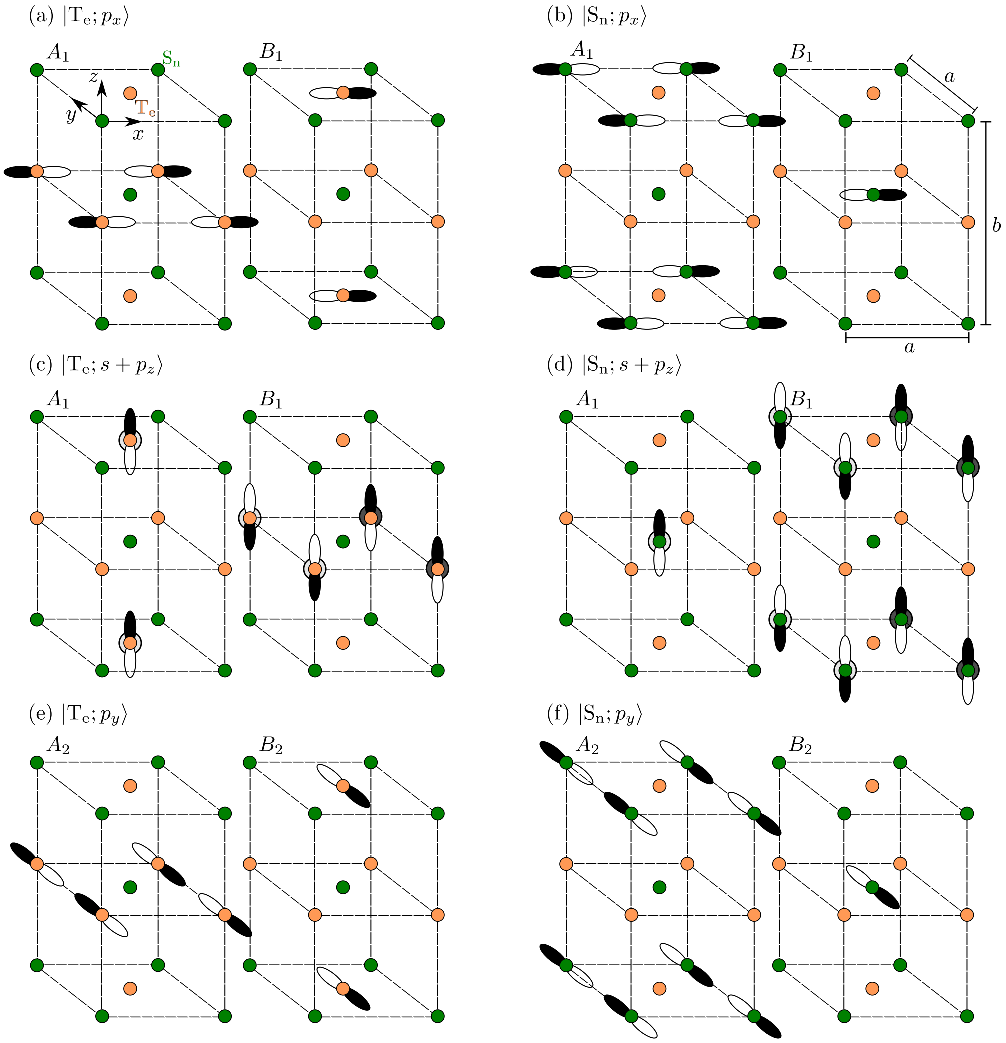


**Figure 1.** Arrangement of the $s$, $p_x$, $p_y$, and $p_z$ orbitals of Sn and Te on the unit cell and their respective IRREPs of $C_{2v}$ for $\boldsymbol{k}$ around $\overline{\mathrm{X}}$. The system of coordinates are indicated in panel (a). A unit cell translation along $x$ introduces a Bloch phase $\psi(x+a) = e^{ik_x a}\psi(x) = -\psi(x)$, since $k_x = \pi/a$ at $\overline{\mathrm{X}}$.

## Full expansion for the finite stacking

In the main text we have focused on the $\overline{\Gamma}-\overline{\text{X}}$ direction (i.e. $k_y = 0$), which is sufficient to discuss the phase transitions seen in the DFT results. To complement this analysis, here we show the full Hamiltonian written in the basis $\{|A_1^t;\uparrow\rangle, |A_1^t;\downarrow\rangle, |B_1^t;\uparrow\rangle, |B_1^t;\downarrow\rangle, |A_1^b;\uparrow\rangle, |A_1^b;\downarrow\rangle, |B_1^b;\uparrow\rangle, |B_1^b;\downarrow\rangle\}$. In general, we can write the Hamiltonian as

$$H = \begin{pmatrix} H_t & V \\ V^\dagger & H_b \end{pmatrix}, \tag{1}$$

where $H_{t/b}$ the top and bottom surfaces solutions for the semi-infinite case, and $V$ is the hybridization matrix. For both symmorphic and nonsymmorphic cases the $H_t$ is the same, since it must match the semi-infinite limit. It reads

$$H_t^{S/NS}(\boldsymbol{k}) = \begin{pmatrix} \Delta_0 & 0 & 0 & \Delta_1 \\ 0 & \Delta_0 & -\Delta_1 & 0 \\ 0 & -\Delta_1 & -\Delta_0 & 0 \\ \Delta_1 & 0 & 0 & -\Delta_0 \end{pmatrix} + \begin{pmatrix} 0 & i\alpha_x & i\gamma_x & 0 \\ -i\alpha_x & 0 & 0 & i\gamma_x \\ -i\gamma_x & 0 & 0 & i\beta_x \\ 0 & -i\gamma_x & -i\beta_x & 0 \end{pmatrix} k_x + \begin{pmatrix} 0 & \alpha_y & \gamma_y & 0 \\ \alpha_y & 0 & 0 & -\gamma_y \\ \gamma_y & 0 & 0 & \beta_y \\ 0 & -\gamma_y & \beta_y & 0 \end{pmatrix} k_y. \tag{2}$$

The bottom surface Hamiltonian can be obtained applying the inversion operator to $H_t(\boldsymbol{k})$. For the symmorphic case it reads $H_b^S(\boldsymbol{k}) = I \cdot H_t(\boldsymbol{k}) = H_t(-\boldsymbol{k})$, since the inversion $I$ simply flips the $t/b$ subspace. In contrast, the nonsymmorphic inversion $\{I, \vec{\ell}\}$ also flips the $A_1/B_1$ subspace, which yields

$$H_b^{NS}(\boldsymbol{k}) = \begin{pmatrix} -\Delta_0 & 0 & 0 & -\Delta_1 \\ 0 & -\Delta_0 & +\Delta_1 & 0 \\ 0 & +\Delta_1 & \Delta_0 & 0 \\ -\Delta_1 & 0 & 0 & \Delta_0 \end{pmatrix} + \begin{pmatrix} 0 & -i\beta_x & i\gamma_x & 0 \\ i\beta_x & 0 & 0 & i\gamma_x \\ -i\gamma_x & 0 & 0 & -i\alpha_x \\ 0 & -i\gamma_x & i\alpha_x & 0 \end{pmatrix} k_x + \begin{pmatrix} 0 & -\beta_y & -\gamma_y & 0 \\ -\beta_y & 0 & 0 & \gamma_y \\ -\gamma_y & 0 & 0 & -\alpha_y \\ 0 & \gamma_y & -\alpha_y & 0 \end{pmatrix} k_y. \tag{3}$$

For convenience and simplicity, in the main text we have neglected $\Delta_1$ and $\gamma_x$ to introduce a simpler picture of the hybridizations. Similarly, the finite $\boldsymbol{k}$ couplings $\mu_{(1|2|3|4|5|6)}$ below are also neglected in the main text since they only contribute to a fine tuning of the energy dispersion.

The symmorphic and nonsymmorphic cases also differ in the top/bottom surface couplings $V$. For the symmorphic stacking, $V = V_S$, which contains three hybridization terms $\delta_{(0|1|2)}$ for $\boldsymbol{k} = 0$, plus $\mu$-couplings for finite $\boldsymbol{k}$. These read

$$V_S = \begin{pmatrix} \delta_0 & 0 & 0 & \delta_2 \\ 0 & \delta_0 & \delta_2 & 0 \\ 0 & \delta_2 & \delta_1 & 0 \\ \delta_2 & 0 & 0 & \delta_1 \end{pmatrix} + \begin{pmatrix} 0 & 0 & i\mu_1 & 0 \\ 0 & 0 & 0 & i\mu_1 \\ i\mu_1 & 0 & 0 & 0 \\ 0 & i\mu_1 & 0 & 0 \end{pmatrix} k_x + \begin{pmatrix} 0 & 0 & \mu_2 & 0 \\ 0 & 0 & 0 & -\mu_2 \\ -\mu_2 & 0 & 0 & 0 \\ 0 & \mu_2 & 0 & 0 \end{pmatrix} k_y. \tag{4}$$

In contrast, for the nonsymmorphic case, $V = V_{NS}$ has only a single hybridization term $\delta_3$ for $\boldsymbol{k} = 0$. Considering also the negligible $\mu$-couplings, it reads

$$V_{NS} = \begin{pmatrix} \delta_3 & 0 & 0 & 0 \\ 0 & \delta_3 & 0 & 0 \\ 0 & 0 & \delta_3 & 0 \\ 0 & 0 & 0 & \delta_3 \end{pmatrix} + \begin{pmatrix} 0 & i\mu_3 & i\mu_4 & 0 \\ -i\mu_3 & 0 & 0 & i\mu_4 \\ i\mu_5 & 0 & 0 & -i\mu_3 \\ 0 & i\mu_5 & i\mu_3 & 0 \end{pmatrix} k_x + \begin{pmatrix} 0 & \mu_6 & 0 & 0 \\ \mu_6 & 0 & 0 & 0 \\ 0 & 0 & 0 & -\mu_6 \\ 0 & 0 & -\mu_6 & 0 \end{pmatrix} k_y. \tag{5}$$

## Edge states

In the main text we have seen that as the number $N$ of [001]-stacked monolayers is reduced, a topological phase transition occurs around $N = 11$ for the odd $N$ stackings (symmorphic), while there is no phase transition for the even $N$ case (nonsymmorphic). Since the stacking is a two-dimensional (2D) system (i.e. it has two periodic directions), one can expect that the mirror Chern number, associated with its (top/bottom) surface states, indicate its topological character in terms of edge states running along its confined side surfaces. Therefore, it indicates if the stacking is a trivial insulator, or a 2D TCI with edge states.

The characteristics of these edge states must depend on the confined directions (e.g. [010] or [110]), and on its atomistic terminations. For the monolayer case, a similar analysis was presented by us in Ref. 1. The generalization of this analysis to the

present [001]-stacking case demands further work due to the more complex structure of the stacking, which goes beyond the scope of this paper. Nevertheless, here we use the effective model to illustrate the effects of further confining the [001] stacking along the [010] direction, which is the simples case (i.e. the [110] confinement would project the $\overline{\mathrm{X}}$ and $\overline{\mathrm{Y}}$ points of the 2D Brillouin zone into a common $\overline{\mathrm{X}}'$ point of the 1D Brillouin zone).

Namely, consider our 2D effective $H$ in Eq. (1), which models either the symmorphic (S) or nonsymmorphic (NS) stacking with $N$ monolayers. For simplicity, we assume that the only $N$-dependent parameters are the $\delta_j$ surface hybridization couplings, which grow as $N$ is reduced. Next, replace $k_y \to -i\partial_y$ to implement the confinement along $y \parallel [010]$, for which we use the finite differences method as discussed in Ref. 2. For the numerical simulation we consider the width $L_y \approx 1000$ Å, discretized into $\sim 100$ lattice points. For the symmorphic stacking we use $\delta_0 = \delta_1 = 10$ meV to represent the $N < 11$ case, and $\delta_0 = \delta_1 = 70$ meV for $N > 11$, and in both cases $\delta_2 = -20$ meV. For the nonsymmorphic stacking, $\delta_3 = -20$ meV. The resulting energy dispersions as a function of $k_x$ are shown in Fig. 2. The parameters chosen to illustrate the $N > 11$ symmorphic case show an in-gap Dirac crossing that is doubly degenerate (localized at opposite edges, $y = \pm L_y/2$). Increasing the hybridization term $\delta_0 = \delta_1$, the $N < 11$ case becomes trivial, which is a consequence of th band inversion discussed in the main text. In contrast, for the nonsymmorphic case there is no phase transition, and in-gap edge states are seen for any value of $\delta_3 < 0$.

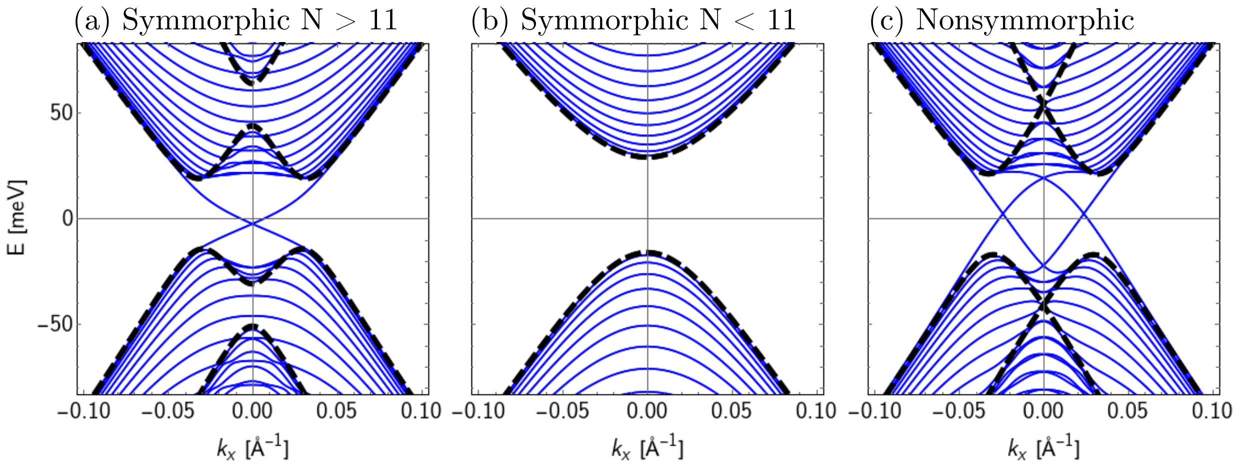


**Figure 2.** Band structure $E(k_x)$ numerically obtained from the effective model for a finite stacking of $N$ monolayers along $z \parallel [001]$, and further confined along $y \parallel [010]$ with a width $L_y$. The thick black dashed lines are band edges from the stacking model ($L_y \to \infty$ and $k_y = 0$), and the blue lines are the quantized confined bands. (a)-(b) For the symmorphic case, the parameters for $N < 11$ and $N > 11$ cases illustrate the topological phase transition by (a) the presence, or (b) the absence, of in-gap 1D topological states (doubly degenerate). (c) For the nonsymmorphic case there is no phase transition and only one case occurs, which shows two pairs of edge states.

It is important to notice that here we have chosen $\delta_2 < 0$ and $\delta_3 < 0$ for the symmorphic and nonsymmorphic cases, respectively. These parameters control similar hybridizations, as discussed in the main text. This choice does not affect the results of Fig. 2(a)-(b) for the symmorphic case, which shows only minor quantitative differences for $\delta_2 > 0$. This shows that the topological nature of the symmorphic case is controlled by the band inversion induced by the $\delta_0$, and $\delta_1$ terms as discussed in the main text. In contrast, for $\delta_3 > 0$, the nonsymmorphic case of Fig. 2(c) would show no edge states. Therefore, the sign of $\delta_3$ defines the topological character of the nonsymmorphic stacking. However, one should expect that as $N$ is reduced, the absolute value of the hybridization $|\delta_3|$ increases, while its sign does not change. Therefore, the nonsymmorphic case must always show ($\delta_3 < 0$), or never show ($\delta_3 > 0$) edge states. Assuming that for large $N$ the symmorphic and nonsymmorphic cases should be similar, here we choose $\delta_3 < 0$. Nonetheless, these parameters and conditions do require further investigations in future works.

## Double Group Character Tables

For convenience, we list here the character tables of the groups mentioned in main text.

First for the bulk SnTe, the little group near the L point of the Brillouin zone is the P$\bar{3}$1m (or $\mathrm{D}_{3\mathrm{d}}^1$). Its character table is shown in Tab. 1. The conduction and valence bands are composed by the $L_6^{\pm}$ double group IRREPs, which can be distinguished by their inversion eigenvalues $\pm 2$.

For the semi-infinite model, the surface states around the $\overline{\mathrm{X}}$ point obey the Pmm2 (or $\mathrm{C}_{2\mathrm{v}}^1$) space group. From the characters table shown in Tab. 2, we extract the characters from the $A_1$ and $B_1$ IRREPs that define the spinless orbitals shown in Fig. 1.

The symmorphic stacking around the $\overline{\mathrm{X}}$ point is characterized by the group Pmmm (or $\mathrm{D}_{2\mathrm{h}}^1$) and the character table shown in Tab. 3. Both double group IRREPS, $X_1$ and $X_2$, have order 2, which implies twofold degeneracy. This matches the hybridization picture, where a fourfold degeneracy at $\overline{\mathrm{X}}$ opens as the number of layers $N$ is reduced.

For the nonsymmorphic stacking, the character table corresponding to the group Pmmn (or $\mathrm{D}_{2\mathrm{h}}^{13}$) is shown in Tab. 4. Since we are interested in the $\overline{\mathrm{X}}$ point ($\boldsymbol{k} = \frac{\pi}{a}\hat{x}$), the allowed IRREPs must satisfy $\chi^\Gamma(E^{2\ell}) = -\chi^\Gamma(E^0)$, which selects the single group IRREPs $\Gamma_5$ and $\Gamma_{10}$, and the double group IRREPs $\Gamma_{13}$ and $\Gamma_{14}$. All of them have order 2. Notice, however, that $\Gamma_{13}$ and $\Gamma_{14}$ form a complex conjugate pair that are combined via time-reversal symmetry to form a single fourth-order IRREP $\Gamma_{13} \oplus \Gamma_{14}$, which gives the fourfold degeneracy for the nonsymmorphic stacking of arbitrary $N$.

**Table 1.** Character Table for the P$\bar{3}$1m (or $\mathrm{D}_{3\mathrm{d}}^1$) double group. This is the space group for the L point in bulk SnTe.

| | $E$ | $\bar{E}$ | $2C_3$ | $2\bar{C}_3$ | $2C_2'$ | $2\bar{C}_2'$ | $I$ | $\bar{I}$ | $2S_6$ | $2\bar{S}_6$ | $3\sigma_d$ | $3\bar{\sigma}_d$ |
|---|---|---|---|---|---|---|---|---|---|---|---|---|
| $A_{1g}$ | 1 | 1 | 1 | 1 | 1 | 1 | 1 | 1 | 1 | 1 | 1 | 1 |
| $A_{2g}$ | 1 | 1 | 1 | 1 | -1 | -1 | 1 | 1 | 1 | 1 | -1 | -1 |
| $E_g$ | 2 | 2 | -1 | -1 | 0 | 0 | 2 | 2 | -1 | -1 | 0 | 0 |
| $A_{1u}$ | 1 | 1 | 1 | 1 | 1 | 1 | -1 | -1 | -1 | -1 | -1 | -1 |
| $A_{2u}$ | 1 | 1 | 1 | 1 | -1 | -1 | -1 | -1 | -1 | -1 | 1 | 1 |
| $E_u$ | 2 | 1 | -1 | -1 | 0 | 0 | -2 | -2 | 1 | 1 | 0 | 0 |
| $L_6^+$ | 2 | -2 | 1 | -1 | 0 | 0 | 2 | -2 | 1 | -1 | 0 | 0 |
| $L_4^+$ | 1 | -1 | -1 | 1 | $i$ | $-i$ | 1 | -1 | -1 | 1 | $i$ | $-i$ |
| $L_5^+$ | 1 | -1 | -1 | 1 | $-i$ | $i$ | 1 | -1 | -1 | 1 | $-i$ | $i$ |
| $L_6^-$ | 2 | -2 | 1 | -1 | 0 | 0 | -2 | 2 | -1 | 1 | 0 | 0 |
| $L_4^-$ | 1 | -1 | -1 | 1 | $-i$ | $i$ | -1 | 1 | 1 | -1 | $i$ | $-i$ |
| $L_5^-$ | 1 | -1 | -1 | 1 | $i$ | $-i$ | -1 | 1 | 1 | -1 | $-i$ | $i$ |

**Table 2.** Character Table for the Pmm2 (or $\mathrm{C}_{2\mathrm{v}}^1$) double group, which is used to describe the semi-infinite model. Here, $C_2 \equiv C_2(z)$, $M \equiv M_y$, and $M' \equiv M_x$.

| | $E$ | $\bar{E}$ | $C_2$ $\bar{C}_2$ | $M$ $\bar{M}$ | $M'$ $\bar{M}'$ |
|---|---|---|---|---|---|
| $A_1$ | 1 | 1 | 1 | 1 | 1 |
| $A_2$ | 1 | 1 | 1 | -1 | -1 |
| $B_1$ | 1 | 1 | -1 | 1 | -1 |
| $B_2$ | 1 | 1 | -1 | -1 | 1 |
| $\Delta_5$ | 2 | -2 | 0 | 0 | 0 |

**Table 3.** Character Table for the Pmmm (or $D^1_{2h}$) double group. Complementing the notation from Tab. 2, here $C_2' \equiv C_2(x)$, $C_2'' \equiv C_2(y)$, $M'' \equiv M_z$, and $I$ is the inversion.

| | $E$ | $\bar{E}$ | $C_2$<br>$\bar{C}_2$ | $C_2'$<br>$\bar{C}_2'$ | $C_2''$<br>$\bar{C}_2''$ | $I$ | $\bar{I}$ | $M$<br>$\bar{M}$ | $M'$<br>$\bar{M}'$ | $M''$<br>$\bar{M}''$ |
|---|---|---|---|---|---|---|---|---|---|---|
| $A_g$ | 1 | 1 | 1 | 1 | 1 | 1 | 1 | 1 | 1 | 1 |
| $B_{1g}$ | 1 | 1 | -1 | 1 | -1 | 1 | 1 | -1 | 1 | -1 |
| $B_{2g}$ | 1 | 1 | 1 | -1 | -1 | 1 | 1 | 1 | -1 | -1 |
| $B_{3g}$ | 1 | 1 | -1 | -1 | 1 | 1 | 1 | -1 | -1 | 1 |
| $A_u$ | 1 | 1 | 1 | 1 | 1 | -1 | -1 | -1 | -1 | -1 |
| $B_{1u}$ | 1 | 1 | -1 | 1 | -1 | -1 | -1 | 1 | -1 | 1 |
| $B_{2u}$ | 1 | 1 | 1 | -1 | -1 | -1 | -1 | -1 | 1 | 1 |
| $B_{3u}$ | 1 | 1 | -1 | -1 | 1 | -1 | -1 | 1 | 1 | -1 |
| $X_1$ | 2 | -2 | 0 | 0 | 0 | 2 | -2 | 0 | 0 | 0 |
| $X_2$ | 2 | -2 | 0 | 0 | 0 | -2 | 2 | 0 | 0 | 0 |

**Table 4.** Character Table for the Pmmn (or $D^{13}_{2h}$) nonsymmorphic double group. For convenience we use a shorthand notation for the nonsymmorphic operations that can be translated to the Seitz notation as: $R^{n\ell} = \{R, n\vec{\ell}\}$, where $R$ labels the point group operations.

| | $E^0$ | $\bar{E}^0$ | $C^\ell_{2x}$<br>$\bar{C}^\ell_{2x}$<br>$C^{3\ell}_{2x}$<br>$\bar{C}^{3\ell}_{2x}$ | $E^{2\ell}$ | $\bar{E}^{2\ell}$ | $C^0_{2z}$<br>$\bar{C}^0_{2z}$<br>$C^{2\ell}_{2z}$<br>$\bar{C}^{2\ell}_{2z}$ | $C^\ell_{2y}$<br>$\bar{C}^{3\ell}_{2y}$ | $\bar{C}^\ell_{2y}$<br>$C^{3\ell}_{2y}$ | $M^0_y$<br>$\bar{M}^0_y$ | $M^\ell_z$<br>$\bar{M}^\ell_z$<br>$M^{3\ell}_z$<br>$\bar{M}^{3\ell}_z$ | $M^{2\ell}_y$<br>$\bar{M}^{2\ell}_y$ | $M^0_x$<br>$\bar{M}^0_x$<br>$M^{2\ell}_x$<br>$\bar{M}^{2\ell}_x$ | $I^\ell$<br>$I^{3\ell}$ | $\bar{I}^\ell$<br>$\bar{I}^{3\ell}$ |
|---|---|---|---|---|---|---|---|---|---|---|---|---|---|---|
| $\Gamma_1$ | 1 | 1 | 1 | 1 | 1 | 1 | 1 | 1 | 1 | 1 | 1 | 1 | 1 | 1 |
| $\Gamma_2$ | 1 | 1 | 1 | 1 | 1 | -1 | -1 | -1 | 1 | 1 | 1 | -1 | -1 | -1 |
| $\Gamma_3$ | 1 | 1 | -1 | 1 | 1 | 1 | -1 | -1 | 1 | -1 | 1 | 1 | -1 | -1 |
| $\Gamma_4$ | 1 | 1 | -1 | 1 | 1 | -1 | 1 | 1 | 1 | -1 | 1 | -1 | 1 | 1 |
| $\Gamma_5$ | 2 | 2 | 0 | -2 | -2 | 0 | 0 | 0 | 2 | 0 | -2 | 0 | 0 | 0 |
| $\Gamma_6$ | 1 | 1 | 1 | 1 | 1 | 1 | 1 | 1 | -1 | -1 | -1 | -1 | -1 | -1 |
| $\Gamma_7$ | 1 | 1 | 1 | 1 | 1 | -1 | -1 | -1 | -1 | -1 | -1 | 1 | 1 | 1 |
| $\Gamma_8$ | 1 | 1 | -1 | 1 | 1 | 1 | -1 | -1 | -1 | 1 | -1 | -1 | 1 | 1 |
| $\Gamma_9$ | 1 | 1 | -1 | 1 | 1 | -1 | 1 | 1 | -1 | 1 | -1 | 1 | -1 | -1 |
| $\Gamma_{10}$ | 2 | 2 | 0 | -2 | -2 | 0 | 0 | 0 | -2 | 0 | 2 | 0 | 0 | 0 |
| $\Gamma_{11}$ | 2 | -2 | 0 | 2 | -2 | 0 | 0 | 0 | 0 | 0 | 0 | 0 | 2 | -2 |
| $\Gamma_{12}$ | 2 | -2 | 0 | 2 | -2 | 0 | 0 | 0 | 0 | 0 | 0 | 0 | -2 | 2 |
| $\Gamma_{13}$ | 2 | -2 | 0 | -2 | 2 | 0 | $2i$ | $-2i$ | 0 | 0 | 0 | 0 | 0 | 0 |
| $\Gamma_{14}$ | 2 | -2 | 0 | -2 | 2 | 0 | $-2i$ | $2i$ | 0 | 0 | 0 | 0 | 0 | 0 |

## References


1. Araújo, A. L., Wrasse, E. O., Ferreira, G. J. & Schmidt, T. M. Topological nonsymmorphic ribbons out of symmorphic bulk. *Phys. Rev. B* **93**, 161101 (2016).
2. Messias de Resende, B., de Lima, F. C., Miwa, R. H., Vernek, E. & Ferreira, G. J. Confinement and fermion doubling problem in Dirac-like Hamiltonians. *Phys. Rev. B* **96**, 161113 (2017).
